# AlphaFold developers Demis Hassabis and John Jumper share the 2023 Albert Lasker Basic Medical Research Award

**DOI:** 10.1172/JCI174915

**Published:** 2023-09-21

**Authors:** Tobin R. Sosnick

**Affiliations:** Department of Biochemistry and Molecular Biology, Pritzker School of Molecular Engineering, Institute for Biophysical Dynamics, University of Chicago, Chicago, Illinois, USA.

The 2023 Albert Lasker Basic Medical Research Award has been given to Demis Hassabis and John Jumper of DeepMind for the invention of AlphaFold, the artificial intelligence (AI) system that solved the long-standing challenge of predicting the three-dimensional (3D) structure of proteins from the one-dimensional (1D) sequence of their amino acids.

The expression “I alphafolded it” is now something I hear almost every day, whether in the lab, during a thesis defense, or at a scientific conference. This transformation of a noun into a verb, akin to the phrase, “I googled it,” mirrors the revolution that has occurred in biological sciences over the past two and a half years. This change began with the announcement on November 30, 2020 (1), of the success of DeepMind’s AI program AlphaFold, which was developed by a team run by Drs. Hassabis and Jumper (2). They essentially solved Nobel Prize winner Christian Anfinsen’s six-decade-old grand challenge in biophysics — the prediction of the structure of a protein based solely on its primary amino acid sequence (3). The full impact of this development began when AlphaFold and similar programs (4) were made widely accessible in July 2021, fundamentally altering the landscape of biological research.

Knowledge of a protein’s atomic level structure is essential for a meaningful understanding of how it functions. Since virtually every biological process involves proteins, this knowledge is critical. These biomolecules can be enzymes, antibodies, or motors found in our muscles, participating in signaling, sensing, motility, immune response, translation, compartmentalization, and regulation. However, the determination of even a single protein structure can be highly laborious. The AI-based breakthrough of providing millions of structures with an accuracy likely comparable to that of experimental methods (5) greatly advances scientific and medical studies on numerous fronts, including areas ranging from drug discovery to protein design and engineering. The paradigm-changing shift is the expectation that an AI-generated structure is available and likely to be as accurate as an experimental structure or at least accurate enough that the prediction can be applied to interpret data or influence further research.

## Solving a fundamental challenge

Not surprisingly, considerable effort has been put into developing prediction methods for protein structure. To this end, John Moult and Krzysztof Fidelis founded the Critical Assessment of Structure Prediction (CASP) in 1994 (6). CASP’s approach, which balanced cooperativity with competition, provided a rigorous, blind format where the field could test different methods. More than 100 research groups would participate in each biannual round of CASP, resulting in considerable advances. However, obtaining a reliable atomic level structure for all but the smallest proteins typically required the knowledge of a structure of a structurally similar (homologous) protein acting as a template.

Results (once corrected for a larger database) largely plateaued by the 11th round of CASP in 2014 (referred to as “CASP11”) (7). Earlier, it had been recognized that by mining the multiple sequence alignments (MSAs) for correlations between residues that are far apart in the 1D sequence, one could identify residues that are likely to be near one another spatially (8). In CASP11, David Baker’s lab employed this strategy to accurately predict a structure without using a template (7). In CASP12, this strategy (along with other improvements) resulted in a noticeable increase in accuracy (9). While impressive, the methods still did not have the accuracy to replace experimental structure determination. However, this progress highlighted that tremendous information content exists in the sequence correlations, and millions of sequences were becoming available through large-scale sequencing projects that allowed for the creation of accurate MSAs (10). As a result, high-accuracy structure prediction appeared achievable if augmented with sequencing data.

## DeepMind, AI, and protein folding

Meanwhile, Dr. Hassabis, a renowned polymath with expertise in computer science and neuroscience, as well as game design (being an expert game player himself), cofounded DeepMind in 2011. DeepMind developed AlphaGo, an AI program that beat the world Go champion in 2016, an achievement that is considered one of the major successes of AI (11). Part of AlphaGo’s success can be attributed to the fact that it had access to a large data set to train on including thousands of human expert games and then millions of self-play games.

Protein structure prediction similarly incorporated Dr. Hassabis’s three major elements that made it an attractive problem for AI (12). First, protein folding is characterized as a massive search problem. Second, the Protein Data Bank (PDB) provides over 100,000 structures for training (12). Third, success in prediction is easily quantifiable by the similarity between the prediction and the known structure, using specific measures such as backbone root-mean-square deviation (RMSD).

Dr. Jumper joined DeepMind in 2017 to work on structure prediction, utilizing his expertise in protein folding. Previously, Dr. Jumper had been a graduate student at the University of Chicago from 2011 to 2017, where he was coadvised by Karl Freed and me. During his studies, Dr. Jumper developed both an extremely fast and clever molecular dynamics (MD) method and a new machine learning approach for simultaneously training all the energy function parameters (13). In the three years prior to graduate school, he worked for D. E. Shaw Research, a research company recognized for its work in advancing MD hardware for longtime protein simulations.

Dr. Jumper was part of the DeepMind team that participated in CASP13 in 2018. The team, along with other groups, applied AI methods that were becoming increasingly powerful (14). DeepMind’s original version of AlphaFold outperformed the other methods in the template-free category (14), but it did not yet achieve the 1 Å accuracy needed to transform people’s view of protein structure prediction. The DeepMind structure prediction group, now being led by Dr. Jumper, accomplished that feat in CASP14 in 2020 (1).

## Success involved multiple factors

To achieve this success, they developed a whole new AI architecture, recognizing that they were predicting protein structures going from a 1D sequence to a 3D physical structure (2, 15). Their approach, along with the inclusion of physics and evolutionary information, was designed into AlphaFold at many stages. Rather than operating a one-directional pipeline, the various algorithmic steps communicate back and forth with each other; for example, an MSA is used to help generate an initial model, which then is used to improve the MSA, which then improves the model, in an iterative manner.

Other factors contributed to AlphaFold’s success. A one-by-one deletion of the various elements in the prediction pipeline produced only a slight decrease in the performance (2). Surprisingly, even the lack of a template had a minimal effect on performance. However, accuracy decreased notably when the MSA was composed of less than approximately 30 sequences. Overall, it has been suggested that AlphaFold was approximately 5–6 years ahead of existing methods (16).

While the physical principles of protein structure are included throughout AlphaFold, there are instances where biophysics was sacrificed for expediency (2, 15). Fundamentally, proteins are polymers, which governs many of their biophysical properties. Nevertheless, this property was not required in the early search stages of the pipeline. Rather, the protein starts as a “residue gas” where each amino acid monomer is free to move as an independent unit, with the backbone connectivity being realized only after the units have found their native positions. This procedure enables the residues to quickly obtain their correct spatial location without needing to deal with the chain’s connectivity, a property that can create substantial barriers when the chain tries to pass through itself. This connectivity-free strategy is nonphysical but is highly efficient from the perspective of global optimization.

Very commendably, DeepMind made the AlphaFold code publicly available at the time of the original 2021 publication (2) and created a database that provides open access to more than 200 million structures that essentially covers the space of all folds (5). This has been tremendously beneficial, as every research biologist has instant access to a prediction, structural biologists can run the program themselves, and experts can study the algorithm as well as improve it or add capabilities. This generosity has further increased AlphaFold’s impact and rapid adoption by the research community.

## AlphaFold’s impact

AlphaFold’s influence goes well beyond the prediction of the structure of individual proteins. A potentially more important area is the prediction of protein-protein interactions. Although not designed for this task, AlphaFold was adapted to docking (17) by simply creating a hypothetical single-chain protein with a chain break between the two partners, a technique employed by another highly successful AI method that was developed shortly afterward (4). Not surprisingly, AlphaFold’s performance can be improved when explicitly trained for docking, as seen in “AlphaFold-Multimer” (18). Success in docking prediction is likely due partially to the simultaneous prediction of folding and docking, which helps resolve the induced fit problem (19).

AlphaFold also works on membrane proteins (20), protein design (21), and protein-ligand interactions, which can facilitate drug development (22). AlphaFold provides a very useful residue-level confidence score to assist the user. Fortuitously, regions that are predicted with low confidence can be used to predict intrinsically disordered regions or regions that fold upon binding, both of which often have biological significance (23). AlphaFold can improve experimental structure determination by fitting its atomic level models into low-resolution data, such as those obtained from the nuclear pore complex (24).

While the structure prediction challenge is effectively solved, many aspects of protein folding, such as dynamics and thermodynamics, remain active areas of research (25). It will be fascinating to see how AI methods impact these fields. Researchers may increasingly be faced with the intellectual dilemma of deciding whether they would rather use AI methods and obtain a more accurate solution for a particular problem, or use methods based on physical principles but that may provide (so far) a less accurate answer.

## Concluding remarks

Following the July 2021 online publication of AlphaFold, I sent my colleagues an email with the subject line “Revolution in structural biology” and text “…I believe the impact of this work may rival that of genomics and sequencing as the tools start to be applied to protein-protein interactions, membrane protein structure prediction, protein design, and drug discovery… We shall see.” Two years later, I can confidently say that we have seen, and it is clear: AlphaFold has transformed biological and biomedical research for the better.

## Version 1 09/21/2023

Electronic publication

## Version 2 09/28/2023

Electronic publication
